# Different Doses of *β*-Cryptoxanthin May Secure the Retina from Photooxidative Injury Resulted from Common LED Sources

**DOI:** 10.1155/2021/6672525

**Published:** 2021-02-10

**Authors:** Cemal Orhan, Mehmet Tuzcu, Hasan Gencoglu, Emre Sahin, Nurhan Sahin, Ibrahim Hanifi Ozercan, Tejas Namjoshi, Vandita Srivastava, Abhijeet Morde, Deshanie Rai, Muralidhara Padigaru, Kazim Sahin

**Affiliations:** ^1^Department of Animal Nutrition, Faculty of Veterinary Science, Firat University, Elazig 23119, Turkey; ^2^Division of Biology, Faculty of Science, Firat University, Elazig 23119, Turkey; ^3^Department of Pathology, Faculty of Medicine, Firat University, Elazig 23119, Turkey; ^4^OmniActive Health Technologies, Biotechnology Park, Pune 411057, India; ^5^OmniActive Health Technologies, Wagle Estate, Thane 400604, India; ^6^OmniActive Health Technologies Inc, Morristown, NJ 07960, USA

## Abstract

Retinal damage associated with loss of photoreceptors is a hallmark of eye diseases such as age-related macular degeneration (AMD) and diabetic retinopathy. Potent nutritional antioxidants were previously shown to abate the degenerative process in AMD. *β*-Cryptoxanthin (BCX) is an essential dietary carotenoid with antioxidant, anti-inflammatory, and provitamin A activity. It is a potential candidate for developing intervention strategies to delay the development/progression of AMD. In the current study, the effect of a novel, highly purified BCX oral formulation on the rat retinal damage model was evaluated. Rats were fed with BCX for four weeks at the doses of 2 and 4 mg/kg body weight in the form of highly bioavailable oil suspension, followed by retinal damage by exposing to the bright light-emitting diode (LED) light (750 lux) for 48 hrs. Animals were sacrificed after 48 hours, and eyes and blood samples were collected and analyzed. BCX supplementations (2 and 4 mg/kg) showed improvements in the visual condition as demonstrated by histopathology of the retina and measured parameters such as total retinal thickness and outer nuclear layer thickness. BCX supplementation helped reduce the burden of oxidative stress as seen by decreased serum and retinal tissue levels of malondialdehyde (MDA) and restored the antioxidant enzyme activities in BCX groups. Further, BCX supplementation modulated inflammatory markers (IL-1*β*, IL-6, and NF-*κ*B), apoptotic proteins (Bax, Bcl-2, caspase 3), growth proteins and factors (GAP43, VEGF), glial and neuronal proteins (GFAP, NCAM), and heme oxygenase-1 (HO-1), along with the mitochondrial stress markers (ATF4, ATF6, Grp78, Grp94) in the rat retinal tissue. This study indicates that oral supplementation of BCX exerts a protective effect on light-induced retinal damage in the rats via reducing oxidative stress and inflammation, also protected against mitochondrial DNA damage and cellular death.

## 1. Introduction

Light-induced degeneration of photoreceptor cells and the disease's progression in the age-related macular degeneration (AMD) comprise the oxidative damage and, eventually, visual cell loss, which can be inhibited or decelerated by the contribution of antioxidants [1, 2]. Accumulation of drusen and *β*-amyloid peptides in the subretinal space resulting in inflammation and photoreceptor degeneration is observed in early AMD [3]. Further, oxidative stress, inflammation, cell damage leading to cell death, and mitochondrial DNA damage are cellular processes associated with AMD [4]. However, the crucial photobiological role of ultraviolet lights (UVB and UVA) induced apoptosis and cytotoxic damage in DNA prevention was documented in earlier reports [5, 6]. The retina is one of the tissues that consume the most oxygen in the human body [7]. Oxidative stress and inflammation governed by the activation of nuclear factor kappa B (NF-*κ*B) have been suggested to mediate crucial parts in retinopathy's pathogenesis [8]. Even though light-emitting diode (LED) usage has advantages like low energy expenditure and longer lifetime, concerns have been raised due to the harmful blue region of the light spectrum from white LEDs, which can cause eventual retinal damage and toxicity because of their intense emission [9].

It has been shown that some carotenoids, such as lutein and zeaxanthin that are located in the macula and retina, have useful properties against oxidative stress and are also protect against photooxidative damage [10–13]. To protect the retinal pigment epithelium against the photooxidative damage, carotenoids are proposed to act through two primary mechanisms: firstly, carotenoids help to filter harmful blue light and, secondly, remove the triple-state molecules, singlet molecular oxygen, and reactive oxygen species such as lipid peroxides and superoxide radical anions [13, 14].

Humans and animals cannot synthesize carotenoids, and they have to get them from dietary origins like fruits and vegetables [15]. Many findings from the various studies have shown that *β*-cryptoxanthin (BCX) has reasonably high bioavailability from its mutual nutritive origins, to the extent that some foods rich in BCX might be equal to or better than *β*-carotene-rich foods as retinol sources [16]. BCX is a provitamin A polar carotenoid (xanthophyll), and its selective uptake and deposition as a macular xanthophyll in the retina and brain are enhanced by specific xanthophyll-binding proteins [17]. Carotenoid oxygenases are suggested to be used by mammals for the retinoid synthesis from provitamin A carotenoids [18]. BCX is thought to be cleaved in the metabolism via the provitamin A-converting enzymes, involving both cytosolic *β*-carotene oxygenase 1 (BCO1) and mitochondrial BCO2, through a multistep process [18, 19].

Comprehending the molecular mechanisms of retinal light injury in the animal models can yield vital information concerning the effects of light in clinical diseases and can be the basis for future treatments to inhibit or delay visual cell loss [20, 21], impaired vision performance, and eye diseases such as AMD. Given that BCX is an efficient provitamin A carotenoid with potent antioxidant effects [16, 22] and in light of the role of vitamin A in eye health and vision performance, including dark adaptation. Hence, it was our main interest to understand if and how BCX can play a role in limiting the mechanistic pathways mediating light-induced retinal injury and photooxidative stress. Therefore, our study measured the effectiveness of BCX using the damaged retinal model on a variety of oxidant and antioxidant biochemical and inflammatory markers. These included interleukin-1*β* (IL-1*β*), interleukin-6 (IL-6), NF-*κ*B; apoptotic proteins; B-cell lymphoma 2 (Bcl-2), Bcl-2-associated X protein (Bax), cysteine-aspartic acid protease-3 (caspase-3); notable proteins and molecules like growth-associated protein 43 (GAP43), glial fibrillary acidic protein (GFAP), neural cell adhesion molecule (NCAM), heme oxygenase-1 (HO-1), vascular endothelial growth factor (VEGF) and the endoplasmic reticulum/mitochondrial stress markers such as activating transcription factor 4 and 6 (ATF4, ATF6), and glucose-regulated proteins 78 and 94 (Grp78, Grp94). Furthermore, we have also evaluated the effects of BCX on the retinal layer pathology, BCX, and retinol changes in the serum and retina of the rats exposed to intense LED illuminance.

## 2. Materials and Methods

### 2.1. Animals and Diets

Twenty-eight Wistar Albino male rats, age: 8 weeks, weight: 180 ± 20 g, were housed in a controlled environment with a 12 : 12-h light-dark cycle at 22°C and were provided with standard rat chow (Table 1) and tap water *ad libitum*. All the experiments were conducted under the National Institutes of Health's Guidelines for the Care and Use of Laboratory Animals and approved by the Firat University Ethics Committee, Elazig (2019-94-143). After providing compliance with the laboratory conditions, rats were randomly divided into four treatment groups, each containing seven animals.

Animals were orally administered with BCX for four weeks followed by 48 hrs (12 hours light/12 hours dark periods) to induce retinal degeneration (RD) with LED light illumination (750 lux), and animals were continued on BCX supplementation during these two days of light exposure. Before intense light exposure, pupils of the animals were dilated with 1% tropicamide, and after exposure, animals were kept in the dark until evaluation. BCX (BCXcel™) was supplied by OmniActive Health Technologies Pvt. Ltd., Mumbai, India (B.No:141025). BCX concentrate was derived from Paprika oleoresin by solvent extraction and column chromatography. BCX concentrate, which contained about 10–80% by weight total xanthophyll, of which about 75–98% by weight was trans-beta-cryptoxanthin, the remaining including zeaxanthin, trans-capsanthin, beta-carotene, and trace amounts of other carotenoids, derived from oleoresin. The active ingredient (*β*-cryptoxanthin) was analyzed by UV spectrophotometer (Shimadzu UV-1800) and Agilent 1200 HPLC System (Agilent Technologies, Santa Clara, CA, USA). For sample preparation, 100 mg sample was weighed, 30 mL of tetrahydrofuran was added in 100-mL amber-colored volumetric flask and sonicated till sample is dissolved with intermediate shaking. After cooling, a volume of up to 100 mL was made with ethyl acetate (sample stock solution A). Next, the extract was pipetted with 5.0 mL of sample stock solution A into a 25-mL volumetric flask, diluted with the mobile phase, and filtered through a 0.45-micron filter. The sample was then run with an HPLC system using YMC- Carotenoid S5 micron (250 mm x 4.6 mm) column and mobile phase methanol: chloroform 900 : 100 at a flow rate of 1.5 mL/min. Chromatograms were examined at 451 nm, and the injection volume was 20 *μ*l. Mixed tocopherol (0.3%) was added to the oil suspension as an antioxidant.

### 2.2. Experimental Design

The experimental design of the groups is summarized in Table 2. The rats were divided into four groups: (i) standard control: these animals only received standard rat chow and tap water ad until the end of the study; (ii) retinal degeneration (RD): fed as the control group, but remained under intense LED lighting for 48 hours after four weeks of the study; (iii) *β*-cryptoxanthin 1 (BCX1): fed as the control group and given BCX intragastrically by oral gavage (2 mg/kg BW/day) then remained under intense LED lighting for 48 hours after the four weeks of the study; (iv) *β*-cryptoxanthin 2 (BCX2): fed as the control group and given BCX intragastrically by oral gavage (4 mg/kg BW/day) then remained under intense LED lighting for 48 hours after the four weeks of the study. The doses of BCX were chosen from a previous study (Sahin et al. 2017). BCX application was also continued during the 48 hours with intensive lighting. Oral gavage is commonly used to deliver substances to animals in pharmacological and toxicological studies [23]. To ensure standardized dosage and to prevent inadvertent oxidation, delivery of the treatment (BCX or corn oil) was administered by oral gavage. Oral gavage was carried out by using a curved metal needle with a bulbed tip. All gavage procedures were carried out once per day at a similar time of day without administering any anesthesia (between 8.30 a.m. and 10 : 30 a.m.) for 6 d per week over four consecutive weeks. BCX was dissolved in corn oil. The control group received corn oil in a similar condition to the treatment of other experimental groups.

For an intense light exposure experiment, each rat was kept in an isolated cage. White light-emitting diodes were placed on the top of the shelves and were measured at a distance of 20 cm from the remote source to achieve 750 lux standard indoor lighting levels for all groups [24]. To allow retinal degeneration to occur, animals were placed in an environmentally organized light stress box equipped with 750 lux of diffuse white LEDs fixed on the inner upper surface, which lightened the inner walls. Animals were sacrificed by cervical dislocation under xylazine (10 mg/kg, i.m.) and ketamine (50 mg/kg, i.m.) anesthesia immediately following the light and dark exposures; eyes were removed and blood tissue collected.

### 2.3. Biochemical Analysis

Immediately after collecting the target samples, blood tissue was centrifuged at 5000 rpm for obtaining the serum. Serum biochemical parameters (glucose, BUN, and ALT and AST) were examined with a biochemistry analyzer (Samsung Co., Suwon, Korea). Tissues were rinsed with 0.5 mL of a phosphate buffer saline solution (pH 7.4). Tissues were then homogenized in 5-10 mL of cold buffer [20 mM HEPES buffer, pH 7.2 containing 1 mM EGTA, 210 mM mannitol and 70 mM sucrose for superoxide dismutase (SOD), 50 nM potassium phosphate, pH 7.0, containing 1 mM EDTA for catalase (CAT), and 50 mM Tris-HCI, pH 7.5, 5 mM EDTA, and 1 mM DTT for glutathione peroxidase (GSH-Px)] per gram tissue for each enzyme assay. Homogenates were centrifuged at 10,000 × g at 4°C for 15 min for CAT and GSHPx and centrifuged at 1,500 × g for 5 min at 4°C for SOD. Supernatants were then removed and assayed for enzyme activities using the commercial kits (Cayman Chemical, Ann Arbor, MI, USA) depending on the colorimetric methods, which were then measured with a highly sensitive Enzyme-Linked Immunosorbent Assay (ELISA) spectrophotometry reader (ELx800 ™ Absorbance Microplate Reader (BioTek Instruments Winooski VT) according to the manufacturer's procedure (Cayman Chemical, Ann Arbor, MI, USA). The protein concentrations were indicated by the Bradford method using Bradford reagent (Sigma Aldrich, Bradford reagent-B6916-1KT, USA). The intra- and interassay coefficients of variation (CV) for SOD, CAT, and GSH-Px were 3.2% and 3.7%, 3.8% and 9.9%, and 5.7% and 7.2%, respectively.

For MDA analyses, samples were homogenized in a mixture of 0.5 mL of HClO_4_ (0.5 M), 2.5 mL distilled water, and 2[6]-di-tert-butyl-p-cresol (BHT) for precipitating proteins and releasing the MDA bound to the amino groups of proteins and other amino compounds. The samples were then centrifuged at 4500 rpm for 5 min, and supernatants were injected into the HPLC system. For this purpose, an HPLC apparatus of Shimadzu UV–vis SPD-10 AVP detector, a CTO-10 AS VP column, and 30 mM KH_2_PO_4_ and methanol (82.5: 17.5, v/v, pH 3.6) at a flow rate of 1 mL/min were used (Shimadzu, Kyoto Japan). Chromatograms were monitored at 250 nm, and the injection volume was 20 *μ*l [25, 26].

Serum and retinal tissue BCX and retinol levels were also analyzed by using HPLC. Samples were homogenized in 1 mL of cold acetone. Homogenized samples were transferred into polyethylene tubes, and 2 mL ethanol was added to the tubes. After 0.3 mL n-hexane was filled into tubes for extractions, they were centrifuged. This step was repeated three times. N-hexane in tubes was evaporated, and the residues were solved in the mobile phase (methanol : acetonitrile : chloroform; 47 : 42 : 11, *v*/*v*) [25].

### 2.4. Histopathological Analysis

After the eye removal of each rat, the retinas were examined grossly. The tissue was then removed for histological study, washed with plain ordinary saline, immersion-fixed (4% paraformaldehyde and then paraffin), and sectioned into 5-*μ*m thick slices using a microtome. Retinal tissues were stained with hematoxylin and eosin (H&E) and examined using light microscopy. The retinal histology was performed for all the groups with slight modifications as defined earlier [27]. The tissue's midsuperior aspect was examined for all histological analyses through a light microscope at 100x (Olympus, BX51, Japan). Retinal edema damage caused by an intense LED light is graded as follows: (+) mild, (++) moderate, and (+++) rated as severe edema.

### 2.5. Western Blot Analysis

Target proteins were detected via western blotting with slight modifications as previously described in detail [28, 29]. The retinal interleukin-1*β* (IL-1*β*), interleukin-6 (IL-6), NF-*κ*B; apoptotic proteins; B-cell lymphoma 2 (Bcl-2), Bcl-2-associated X protein (Bax), cysteine-aspartic acid protease-3 (caspase-3); notable proteins and molecules like growth-associated protein 43 (GAP43), glial fibrillary acidic protein (GFAP), neural cell adhesion molecule (NCAM), heme oxygenase-1 (HO-1), vascular endothelial growth factor (VEGF) and the endoplasmic reticulum/mitochondrial stress markers such as activating transcription factor 4 and 6 (ATF4, ATF6), and glucose-regulated proteins 78 and 94 (Grp78, Grp94), and actin (as a reference protein to control protein loading) were targeted. Briefly, after the homogenization, each group of animals was examined in triplicates for each experimental situation. 20-50 *μ*g of total proteins was transferred to a nitrocellulose membrane (Schleicher and Schuell Inc., Keene, NH, USA) via immunoblotting after the electrophoresis using Bio-Rad Mini-Protean Tetra electrophoresis “wet-transfer” system (Bio-Rad, California, USA). The phosphorylated form of antibodies against IL-1*β*, IL-6, NF-*κ*B, VEGF, Bax, Bcl2, Caspase-3, GAP43, GFAP, NCAM, HO-1, ATF4, ATF6, Grp78, and Grp94 (Abcam, Cambridge, UK) was diluted in a concentration of (1 : 1000-1 : 2000) in a PBS buffer which contains 0.05% of tween20. The loading of proteins was checked by a monoclonal mouse antibody against *β*-actin (A5316; Sigma). Blots were performed at least three times to confirm the reproducibility of the results. Bands were analyzed densitometrically using an image analysis system (Image J; National Institute of Health, Bethesda, USA).

### 2.6. Statistical Analysis

The sample size of the study was determined as 28 rats for all groups (7 animals per group) with the help of the G ∗ Power package program (Version 3.1.9.2) with alpha error 0.05 and 85% power with an effect size of 0.69 [30]. Conformity to the normality assumption from the prerequisites of the parametric tests was performed using the “Shapiro-Wilk” test. The homogeneity of the variances was checked with the “Levene” test. Analysis of variance (ANOVA) test was performed to determine the differences between the groups, and *post hoc* Tukey test was used for multiple comparisons of the groups. All analyzes performed using the SPSS statistical program (IBM, SPPS Version 21). *p* values < 0.05 were considered significant. Data is presented as mean and standard deviation.

## 3. Results

The effects of BCX1 and BCX2 supplementation on the serum biochemical parameters are presented in Table 3. BCXcel™ supplementation did not change the serum levels of glucose (*p* = 0.890), creatinine (*p* = 0.953), blood urea nitrogen (BUN) (*p* = 0.654), total protein (TP) (*p* = 0.661), albumin (ALB) (*p* = 0.725), globulin (GLOB) (*p* = 0.955), and activities of alanine aminotransferase (ALT) (*p* = 0.698), aspartate aminotransferase (AST) (*p* = 0.866), alkaline phosphatase (ALP) (*p* = 0.892), and total bilirubin (TBIL) level (*p* = 0.101), in all the groups of rats (*p* > 0.05). Changes in serum and retina activities of SOD, GSH-Px, CAT, and MDA, BCX, and retinol levels during intense LED light-induced retinal degeneration between the groups are presented in Table 4. Accordingly, serum and retina MDA levels were the highest in the RD group, while BCX1 and BCX2 administration significantly decreased serum and retinal tissue MDA levels (*p* < 0.0001). Retina SOD, GSHPx, and CAT activities were measured at the highest levels in the control group, while the lowest values were determined in the RD group (*p* < 0.05). However, BCX administration significantly increased antioxidant enzymes' retinal activities, especially the BCX2 dose, which was more effective (*p* < 0.001). Serum and retina BCX levels were not detected in the control and RD groups, while in the BCX2 group, serum levels were 2.3 times, and retinal levels were 2.8 times higher than the BCX1 group (*p* < 0.05). Serum and retina retinol levels were found in the lowest number in the RD groups, whereas BCX2, BCX1, and control groups were found in higher amounts, respectively (*p* < 0.05).

**Figure 1:**
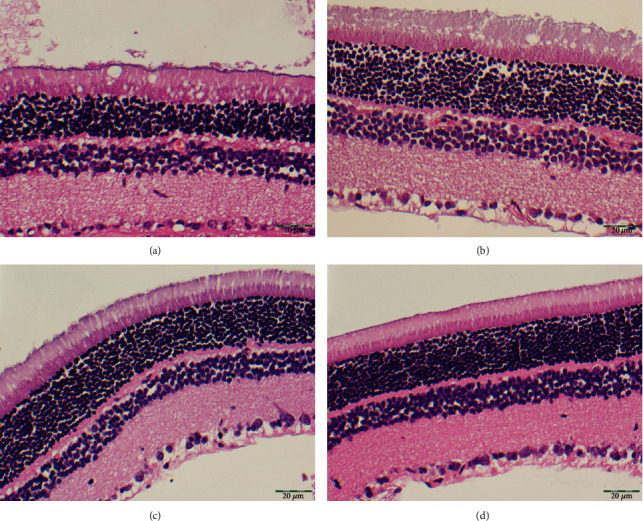
The histopathological appearance of the rat retinas exposed to the intense LED light-induced retinal degeneration between the groups. (a) Control. (b) RD. (c) RD+BCX1. (d) RD+BCX2. (H&E X400), (Bar = 20 *μ*m). BCX: *β*-cryptoxanthin; LED: light-emitting diode; RD: retinal degeneration.

**Figure 2:**
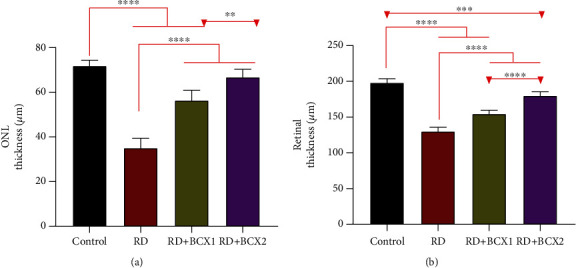
Treatment with *β*-cryptoxanthin (BCX) altered retinal morphology under intense LED light-exposed rats. (a) Quantification of the ONL thickness compared with the control. (b) Quantification of the Retinal thickness compared with the control (*n* = 7; *P* < .05). BCX: *β*-cryptoxanthin; LED: light-emitting diode; ONL: outer nuclear layer; RD: retinal degeneration.

A representative image showing the histopathological effect of both doses of BCXcel™ supplementation on intense LED light-induced retinal degeneration is presented in Figure 1. The retina had a normal histological appearance in the control group. In the RD group, severe edema (+++) was observed in the ganglion layer of the retina, and significant thickening was observed in the outer plexiform layer of the retina. In the RD+BCX1 group, edema decreased in the ganglion layer (++), and there was a slight decrease in the thickening of the outer plexiform layer of the retina. In the RD+BCX2 group, very mild edema (+) near the normal was observed in the ganglion layer, and a marked decrease in the thickening of the outer plexiform layer of the retina was observed.  Figure 2 demonstrates that four weeks of BCXcel™ supplementation significantly preserved the quantifications of both retinal thickness and the ONL thickness of the intense LED light-exposed rats, compared with the control and RD groups (*p* < .05).

**Figure 3:**
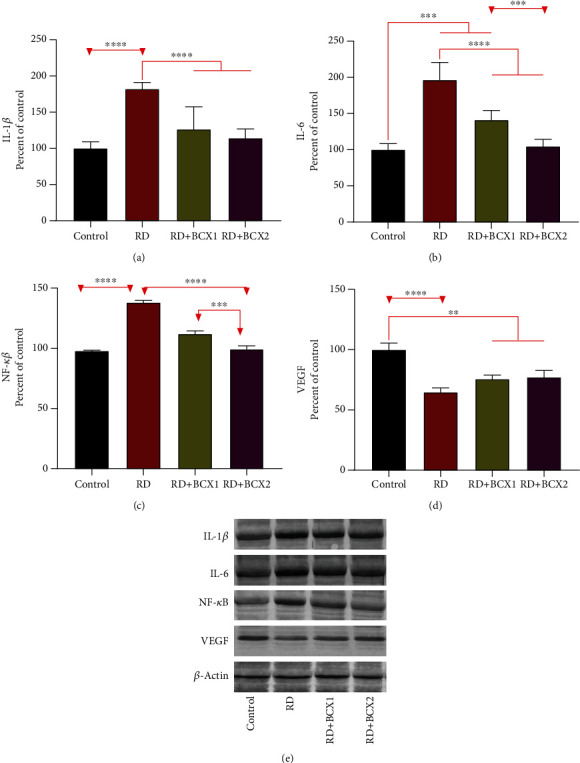
Effects of BCX on retinal protein levels of IL-1*β* (a), IL-6 (b), NF-*κ*B (c), and VEGF (d) levels in rats with RD. The intensity of the western blot bands (e) was quantified by densitometric analysis, and *β*-actin was included to ensure equal protein loading. Data are expressed as a ratio of the control value (set to 100%). The bar represents the standard error of the mean. Blots were repeated at least three times (*n* = 3) and a representative blot is shown. Asterisks indicate statistical differences among groups (^∗∗^*p* < .01; ^∗∗∗^*p* < .001; ^∗∗∗∗^*p* < .0001). BCX: *β*-Cryptoxanthin; IL-1*β*: interleukin-1*β*; IL-6: interleukin-6; NF-*κ*B: nuclear factor kappa B; RD: retinal degeneration; VEGF: vascular endothelial growth factor.

**Figure 4:**
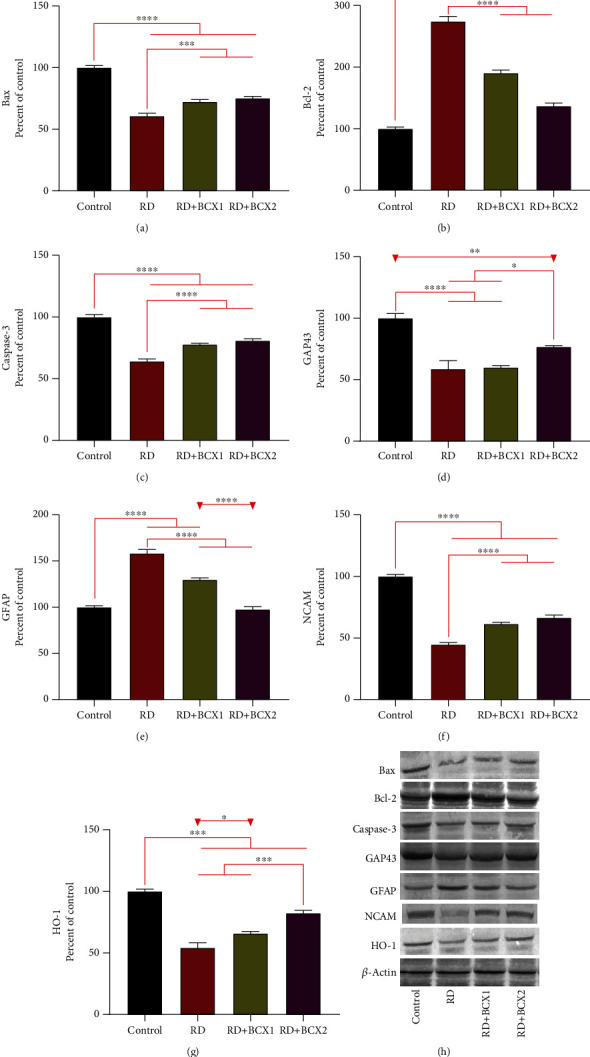
Effects of BCX on retinal protein levels of Bax (a), Bcl-2 (b), Caspase-3 (c), Gap43 (d), GFAP (e), NCAM (f) and HO-1 (g) levels in rats with RD. The intensity of the western blot bands (h) was quantified by densitometric analysis, and *β*-actin was included to ensure equal protein loading. Data are expressed as a ratio of the control value (set to 100%). The bar represents the standard error of the mean. Blots were repeated at least three times (*n* = 3) and a representative blot is shown. Asterisks indicate statistical differences among groups (^∗^*p* < .05; ^∗∗^*p* < .01; ^∗∗∗^*p* < .001; ∗∗∗∗*p* < .0001). BCX: *β*-cryptoxanthin; Bax: Bcl-2-associated X; Bcl-2: B-cell lymphoma 2; Caspase-3: cysteine-aspartic acid protease-3; GAP43: growth-associated protein 43; GFAP: glial fibrillary acidic protein; HO-1: heme oxygenase-1; NCAM: neural cell adhesion molecule; NF-*κ*B: nuclear factor kappa B; RD: retinal degeneration.

**Figure 5:**
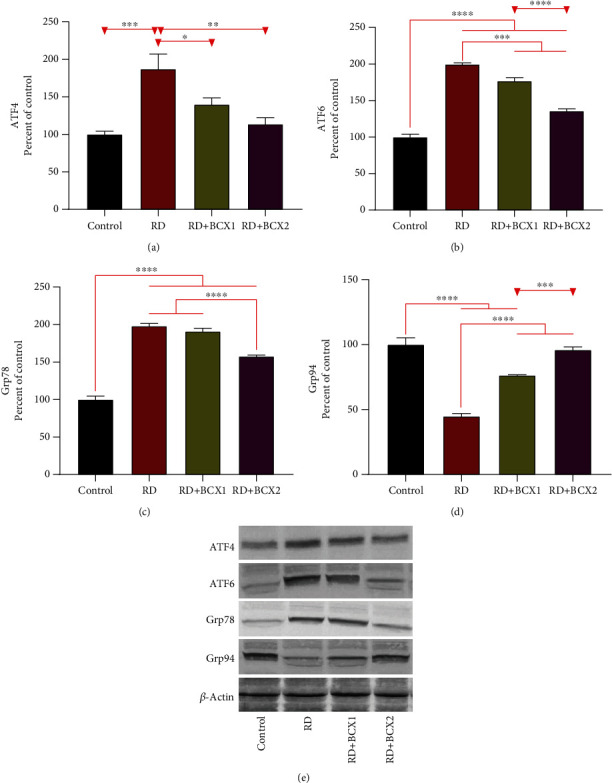
Effects of BCX on retinal protein expression of ATF4 (a), ATF6 (b), Grp78 (c), and Grp94 (d) levels in rats with RD. The intensity of the western blot bands (e) was quantified by densitometric analysis, and *β*-actin was included to ensure equal protein loading. Data are expressed as a ratio of the control value (set to 100%). The bar represents the standard error of the mean. Blots were repeated at least three times (*n* = 3) and a representative blot is shown. Asterisks indicate statistical differences among groups (^∗^*p* < .05; ^∗∗^*p* < .01; ^∗∗∗^*p* < .001; ^∗∗∗∗^*p* < .0001). ATF4: activating transcription factor 4; ATF6: activating transcription factor 4; BCX: *β*-cryptoxanthin; Grp78: glucose-regulated protein 78; Grp94: glucose-regulated protein 94; RD: retinal degeneration.

The effects of both doses of BCXcel™ supplementation on the levels of inflammatory genes, IL-1*β*, IL-6, and NF-*κ*B along with VEGF, are shown in Figure 3. RD group showed significantly more IL-1*β*, IL-6, NF-*κ*B levels in all groups compared to other groups (*p* < 0.001); however, BCXcel™ supplementation significantly decreased these levels while bringing them closer to control levels, especially in the group administered with BCX (*p* < 0.001). Also, retina VEGF levels were found higher in control, while the RD and BCX1/BCX2 groups decreased significantly (*p* < 0.0001).  Figure 4 shows the effects of two doses of BCXcel™ on Bax, Bcl-2, Caspase-3, Gap43, GFAP, NCAM, and HO-1 retinal protein levels in rats. Proapoptotic proteins Bax and Caspase-3 retina levels were upregulated depending on the amount of BCXcel™ supplement (*p* < 0.001), whereas the antiapoptotic Bcl-2 levels were found to be downregulated (*p* < 0.0001). While GAP43 levels decreased significantly in all groups compared to the control group (*p* < 0.01), it was significantly lower in the BCX2 group compared to the RD group (*p* < 0.05). GFAP levels showed the most significant increase in the RD group compared to all groups (*p* < 0.001), while BCX application decreased these levels significantly (*p* < 0.001), and there was no difference between BCX and control (*p* > 0.05). NCAM levels decreased in all groups compared to control (*p* < 0.001), while both BCX doses significantly increased NCAM compared to the RD group (*p* < 0.001). While HO1 levels decreased in all groups compared to control (*p* < 0.001), BCX1 (*p* < 0.05), and BCX2 (*p* < 0.001) significantly increased HO1 levels compared to RD group. The effects of two doses of BCXcel™ on ATF4, ATF6, and Grp78, Grp94 protein levels in rat retinas are shown in Figure 5. Accordingly, ATF4 and ATF6 levels significantly increased in all groups compared to the control group (*p* < 0.001). While this increase was reduced with BCXcel™ supplementation for both proteins, the lowest level was found in the BCX2 group, which was very close to the control (*p* < 0.05). In contrast, Grp78 levels increased in all groups compared to control (*p* < 0.001), and a significant decrease was observed in the BCX2 group when compared to the RD group and even BCX1 group (*p* < 0.001). In contrast, while Grp94 levels decreased significantly compared to control (*p* < 0.001), this decrease was found in the BCX2 group at the lowest level and close to control (*p* < 0.05).

## 4. Discussion

This study aimed to investigate the well-established provitamin A carotenoid, BCX, to limit LED-induced retinal damage, including its adverse consequences on metabolic markers and histopathological changes within the retina.

Reactive oxygen species (ROS) have been reported to cause cellular damage by attacking macromolecules in the visual cells when the intense light exposure time exceeds the threshold [31]. Two types of damage caused by light have earlier been proposed: the first contains rhodopsin and affects the photoreceptors; the second concerns retinal pigment epithelium (RPE), which is selectively vulnerable to high-energy blue light [21]. Excessive LED light exposure could lead to phototoxic effects because of the potent blue light hazard; accordingly, it can damage the macula [9, 32]. However, macular degeneration is the primary cause of blindness and severe visual deterioration of older people over 65 [33].

MDA is a well-established marker of light-induced oxidative stress that is easily detectable in the retina. MDA is produced by photoreactive lipofuscin granules, which generate H_2_O_2_ and other potentially cytotoxic molecules in response to light in RPE [34, 35]. Our study showed similar outcomes to long time constant (200 lux, 2 to 8 days) [36], or short time-intense (3,000 lux, 2 hours) [37], exposure to LED light both showed similar results such as photoreceptor cell deaths, remodeling of the retina and its cellular layers. In the RD group, the serum and retina lipid peroxidation marker, MDA, significantly increased, while the BCXcel™ consumption significantly reversed this effect of lipid peroxidation. This result parallels the findings of a recent study that showed LED exposure critically increases lipid peroxidation and 4-HNE production at the anterior segment level [38].

Our study has also clearly demonstrated that retina SOD, CAT, and GSH-Px activities and serum and retina retinol levels increased with BCXcel™ supplementation. Significant protective effects have also been characterized by both the increase of these BCX levels and the regulation of prominent protein levels as a result of the one-month BCX supplementations to the usual diet, which continued for 48 hours (12 h light/12 h dark) of intense LED light retinal degeneration. Using histopathological measurements, we also determined that retinal thickness, outer nuclear layer (ONL) thickness, and damaged retinal ganglion cell layers, which were degenerated due to exposure to LED light, were preserved through BCXcel™ supplementation at both doses of BCX.

Inhibition of NF-*κ*B activation and downregulation of cellular inflammatory genes such as IL-1 family and IL-6 were proposed as unique retinal protection mechanisms during antioxidant treatments like curcumin [39]. NF-*κ*B is a transcription factor found in all cell types found inactive in the cytoplasm and can move to the nucleus when activated [40]. The IL-1 family is a cluster of 11 cytokines that induce a complex proinflammatory cytokine linkage plus control and initiate inflammatory responses over and done with the expression of integrins on leukocytes and endothelial cells [41]. IL-6 is an interleukin that functions as a proinflammatory cytokine and an anti-inflammatory myokine [42]. Contributors of the IL-1 family facilitate the photoreceptor cell death, inflammation, and angiogenesis in retinal degenerative diseases [41, 43], their reduction besides NF-*κ*B in the retina has been significantly observed in the present study as the BCX supplement increased.

Vascular endothelial growth factor (VEGF) is a signal protein produced by cells that stimulate vasculogenesis and angiogenesis [44]. In this study, VEGF levels decreased in the RD group and did not statistically change with the addition of BCX. However, blue light is known to trigger inflammatory and angiogenic gene expressions in the early stages and has been reported to increase vascular endothelial growth factor (VEGF) secretion in A2E-loaded RPE cells [45]. In our study, if exposure to LED light was prolonged, BCX efficacy at these levels would be more noticeable as a ROS scavenger [46, 47].

We also observed that proapoptotic Bax and caspase-3 levels were decreased significantly in the RD group compared to control, which was reversed by BCX supplementation at both doses. The adverse effects of excessive LED damage on the antiapoptotic marker, Bcl-2, were evident in the RD group but were reversed positively by BCXcel™ supplementation. Photoreceptor cells were found to show intense light-induced apoptosis in chicks and albino fishes' retinas under constant intense light [20, 48]. Similarly to our results, in a recent study in which ganglion cell injury was induced through experimental ocular glaucoma model in rats, it was shown that ocular hypertension provoked apoptosis via the intrinsic pathway, owing to Bax and caspase-3 activation, in both retina and cornea, which also found to led DNA damage due to p53 activation [49]. As a result of antioxidant therapy, there was a suppression of proapoptotic markers and stimulation of antiapoptotic markers of ganglion cell damage, supporting antioxidants' role in reducing retinal damage [49, 50]. Contrary to the findings in the present study, the proapoptotic marker Bax was upregulated in the retina for up to 24 hours after a blast retinal damage study, whereas cytosolic Bax is shown to decrease 3 and 6 hours after retinal artery occlusion injury, while mitochondrial Bax levels rise at 3, 6, and 24 hours, indicating that Bax was settled in the mitochondria [51, 52]. It was shown that degeneration of retinal capillaries under disease conditions could be reduced by antioxidant therapy through caspase-3 and nuclear factor-*κ*B (NF-*κ*B) activation, which plays a vital role in retinal capillary apoptosis [43, 53].

BCX supplementation was able to reverse the 1.5-fold increase in retina GFAP levels measured in the RD group. Indeed, increased GFAP levels have also been reported by de Raad and colleagues [54], who showed that GFAP accumulated in Müller cells in response to photoreceptor damage in the rat retina [54]. GFAP is upregulated during the light stress and blue light exposure, which leads to a nonspecific reactive change of Müller cells known as gliosis [55, 56]. However, GFAP accumulation in Müller cells was reported as an essential photoreceptor stress parameter in retinopathy induced rats, in accordance with our findings [57]. In a recent study, a pattern of GFAP ADPase staining also suggested a robust Müller cells/astrocytes reactivity in the Goto-Kakizaki rat retina [58]. In another novel study, a Chinese traditional antioxidant composition FSR prevented retinal thickness, including the ONL and retinal thickness in the diabetic rat retina, and similarly reduced the GFAP expressions in retinal tissues [59].

In the present study, retinal HO-1 levels decreased in the RD group, while the addition of BCXcel™ to the diet increased the levels similar to the control group. It is inversely proportional to an earlier report that created retinal damage via LED lights of different wavelengths and claimed that the iron ion load increased retinal damage in addition to oxidative stress and accordingly raised HO-1 levels [60]. In other studies similar to ours, HO-1 levels were found to be increased and protected light degeneration in the retina, while the antioxidant phytochemicals supported increased protection of the retina [39, 61].

Activating transcription factor 4 (ATF4) is the main component that encodes the cAMP response element-binding transcription factor, stimulating cell survival via modulation of redox reactions, stress response, protein synthesis, and secretion [62, 63]. ATF4 is triggered by stress signals such as anoxia/hypoxia, amino acid abstinence, endoplasmic reticulum stress, and oxidative stress [62]. Activating transcription factor 6 (ATF6) is a transmembrane protein located in the unfolded protein response in mammals and serves as a sensor for the endoplasmic reticulum homeostasis [64, 65]. ATF6 is necessary to optimize protein folding, secretion, and degradation throughout the ER stress and, therefore, ease the improvement of acute stress and indulgence for chronic stress [66]. In our study, both ATF4 and ATF6 levels increased up to 2 times in the RD group compared to the control, and these levels decreased with BCXcel™ supplementation primarily via the higher dose. These data allude to one of the mechanisms by which BCXcel™ can protect against increased light stress, a commonly described mechanism of light-induced retinal damage [67, 68]. Our findings also support that BCXcel™ supplementation normalized GRP78 and GRP94 levels to similar levels compared to the control group. It is proposed that ATF6 acts in synchronization with GRP78 and affects the response to stress, which is in line with the results obtained in our previous study (S. X. [69]). We also observed the increased levels of ER chaperone GRP94 via higher doses of BCXcel™ supplementation. This finding supported a previous report that BCX could positively modulate the stress response and was consistent with the requirement of maintaining hematopoietic stem cell interactions [70]. BCX has been revealed the upregulation of the energy metabolism, response to stress, reducing inflammation, and protein homeostasis as the primary metabolic objectives of this xanthophyll carotenoid [46].

## 5. Conclusion

**Figure 6:**
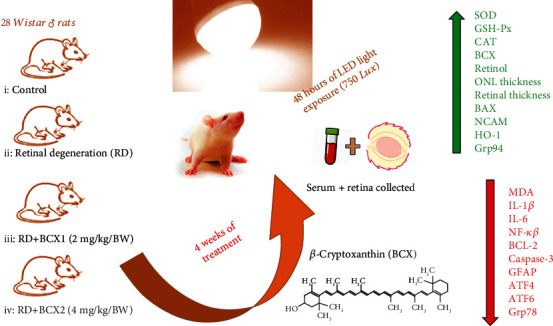
Study overview.

In conclusion, oral supplementation of BCX has effectively demonstrated in our four weeks study that in a rat model, and BCXcel™ has a protective effect on high-intensity light-induced retinal damage by reducing oxidative stress and inflammation as well as protecting against mitochondrial DNA damage and cellular death (Figure 6). Present findings of this study on BCX doses have provided new in vivo evidence of the potential medicinal utilization of BCX in the suppression of diseases connected to intense LED toxicity on the retina and in those in which light has been considered a potential pathological inducer (e.g., AMD). Although MDA is an important oxidative stress marker, measuring MDA alone limits this study. In addition to MDA, measuring 8-isoprostane and 4-hydroxynonenal (4-HNE) for protein oxidation and 8-hydroxy-2′-deoxyguanosine (8-OHdG) levels for DNA/RNA oxidation would further strengthen similar studies. Another limitation is that the animal data cannot be extrapolated directly to humans due to differences between nutrient requirements and the severity and duration of metabolism and dietary therapy. In conclusion, considering that LED lighting has been widely used, it is recommended to conduct more molecular studies using carotenoids as protective agents against the adverse effects of LED.

## Figures and Tables

**Table 1:** Ingredients and chemical analysis of the standard diet.

Ingredients	Quantity (%)
Maize	30.22
Barley	10.07
Soybean meal	38.28
Sunflower seed meal	6.04
Wheat bran	10.08
Molasses	3.02
Limestone	1.51
Salt	0.08
DL-Methionine	0.30
Dicalcium phosphate	0.20
Vitamin-mineral premix ∗	0.20
Chemical analysis	
Crude protein %	24.00
Metabolizable energy (kcal/kg^∗∗^)	3100
Ether extract %	3.40
Crude cellulose %	6.90
Ash %	8.10
Calcium %	1.30
Phosphorus %	0.90

^∗^In per kilogram of vitamin-mineral mixture: 1.8 mg all-trans retinyl acetate (vitamin A), 0.025 mg cholecalciferol (vitamin D), 12.5 mg whole rac-alpha-tocopherol acetate (vitamin E), 1.1 mg menadione sodium bisulfite (vitamin K3), 1.1 mg thiamine (vitamin B1), 4.4 mg riboflavin (vitamin B2), 35 mg niacin (vitamin B3), 10 mg calcium pantothenate (vitamin B5), 2.2 mg vitamin B6, 0.02 vitamin B12, 0.55 mg folic acid, 0.1 mg d-biotin, 40 mg Mn (MnO), 12.5 mg Fe (FeSO4), 25 mg Zn (ZnO), 3.5 mg Cu (CuSO4), 0.3 mg I (KI), 0.15 mg Se (Na2SeO3), 175 mg choline chlorite (C5H14ClNO). ^∗∗^Metabolic energy was calculated according to the National Research Council [71].

**Table 2:** Experimental design of the study.

Groups	Standard control *n* = 7	RD control *n* = 7	RD+BCX1 Dose level 1 *n* = 7	RD+BCX2 Dose level 2 *n* = 7
Dose	—	—	2 mg/kg BW of active *β*-cryptoxanthin	4 mg/kg BW of active *β*-cryptoxanthin
LED illumination	—	Intense LED light (750 lux) Exposure for 48 hours after four weeks	Intense LED light (750 lux) Exposure for 48 hours after four weeks	Intense LED light (750 lux) Exposure for 48 hours after four weeks

BCX: *β*-cryptoxanthin; BW: Body Weight; LED: Light Emitting Diode; RD: Retinal Degeneration.

**Table 3:** Effects of BCX supplementation on serum biochemical parameters in rats exposed to LED light.

Items	Groups				*p* ^∗^
	Control	RD	RD+BCX1	RD+BCX2	
Glucose, mg/dL	119.71 ± 8.12	120.43 ± 8.08	117.71 ± 13.62	122.06 ± 10.92	0.890
Creatinine, mg/dL	0.41 ± 0.11	0.42 ± 0.12	0.39 ± 0.09	0.41 ± 0.14	0.953
BUN, mg/dL	21.96 ± 1.89	22.91 ± 1.38	23.04 ± 2.66	22.34 ± 0.75	0.654
TP, g/dL	7.47 ± 0.77	7.01 ± 0.51	7.30 ± 1.00	7.41 ± 0.55	0.661
ALB, g/dL	3.57 ± 0.34	3.39 ± 0.46	3.49 ± 0.30	3.57 ± 0.28	0.725
GLOB, g/dL	3.76 ± 0.46	3.83 ± 0.47	3.71 ± 0.68	3.69 ± 0.32	0.955
ALT, U/L	71.14 ± 5.64	68.71 ± 4.61	67.86 ± 5.64	72.43 ± 13.25	0.698
AST, U/L	91.29 ± 7.06	96.43 ± 16.30	92.29 ± 9.89	95.71 ± 17.95	0.866
ALP, U/L	132.57 ± 9.50	130.29 ± 14.50	135.57 ± 21.03	137 ± 22.35	0.892
TBIL, mg/dL	0.21 ± 0.02	0.22 ± 0.02	0.23 ± 0.02	0.24 ± 0.03	0.101

BCX: *β*-cryptoxanthin; BUN: blood urea nitrogen; TP: total protein; ALB: albumin; GLOB: globulin; ALT: alanine aminotransferase; AST: aspartate aminotransferase; ALP: alkaline phosphatase; TBIL: total bilirubin; RD: retinal degeneration. Data are presented as means and standard deviations. Means in the same line without a common superscript differ significantly (*p* < .05; ^∗^ANOVA and Tukey's *post hoc* test).

**Table 4:** Effects of BCX supplementation on MDA, BCX, retinol levels, and antioxidant enzyme activities in rats exposed to LED light.

Markers	Groups			
	Control	RD	RD+BCX1	RD+BCX2
Serum MDA, nmol/mL	0.45 ± 0.08^c^	1.19 ± 0.17^a^	0.79 ± 0.12^b^	0.64 ± 0.07^b^
Retina MDA, nmol/mg	0.8 ± 0.11^d^	1.81 ± 0.12^a^	1.51 ± 0.08^b^	1.12 ± 0.15^c^
Retina SOD, U/mg protein	85.34 ± 6.57^a^	56.71 ± 7.11^c^	65.9 ± 4.77^bc^	75.13 ± 6.74^b^
Retina GSH-Px, U/mg protein	29.48 ± 1.83^a^	14.29 ± 1.78^d^	19.16 ± 1.62^c^	23.74 ± 2.23^b^
Retina CAT, U/mg protein	33.96 ± 3.9^a^	13.88 ± 1.5^c^	20.84 ± 3.15^b^	25.2 ± 3.39^b^
Serum BCX, nmol/L	—	—	6.86 ± 1.35^b^	15.83 ± 1.83^a^
Retina BCX, nmol/g	—	—	0.2 ± 0.08^b^	0.56 ± 0.11^a^
Serum retinol, ng/mL	275.49 ± 27.91^ab^	240.43 ± 20.61^b^	282.87 ± 19.57^a^	288.3 ± 27.9^a^
Retina retinol, *μ*g/g	5.69 ± 0.97	4.75 ± 0.81	5.87 ± 1.07	5.92 ± 1.34

The data are presented as means ± standard deviations. Mean values in the same row without a common superscript (a–d) differ significantly (*p* < .05). BCX: *β*-cryptoxanthin; MDA: malondialdehyde; RD: retinal degeneration; SOD: superoxide dismutase; GSH-Px: glutathione peroxidase; CAT: catalase.

## Data Availability

The data used to support the findings of this study are included within the article and the supplementary information files.

## Supplementary Materials

Supplementary Materials Figure 3 S1: (A-E) shows the full immunoblots related to Figure 3. Figure 3 S1: (A-H) shows the full immunoblots related to Figure 3. Figure 3 S1: (A-E) shows the full immunoblots related to Figure 5.
