# Supplementary material for: Different Doses of β-Cryptoxanthin May Secure the Retina from Photooxidative Injury Resulted from Common LED Sources
Source: Oxid Med Cell Longev. 2021 Feb 10;2021:6672525. doi: 10.1155/2021/6672525 (PMC7895591; doi:10.1155/2021/6672525)
Supplement: Supplementary Materials — Figure 3 S1: (A-E) shows the full immunoblots related to Figure 3. Figure 3 S1: (A-H) shows the full immunoblots related to Figure 3. Figure 3 S1: (A-E) shows the full immunoblots related to Figure 5. [file 6672525.f1.docx]

**SUPPLEMENTARY INFORMATION**

**---------------------------------------------**

**Different Doses of β-cryptoxanthin may Secure the Retina from Photo-oxidative Injury Resulted from Common LED Sources**

Cemal Orhan^1^, Mehmet Tuzcu^2^, Hasan Gencoglu^2^, Emre Sahin^1^, Nurhan Sahin^1^, Ibrahim Hanifi Ozercan^3^, Tejas Namjoshi^4^, Vandita Srivastava^4^, Abhijeet Morde^5^, Deshanie Rai^6^, Muralidhara Padigaru^5,^ Kazim Sahin^1,^*

*^1^Department of Animal Nutrition, Faculty of Veterinary Science, Firat University, Elazig, 23119, Turkey*

*^2^Division of Biology, Faculty of Science, Firat University, Elazig, 23119, Turkey*

*^3^Department of Pathology, Faculty of Medicine, Firat University, Elazig, 23119, Turkey*

*^4^OmniActive Health Technologies, Biotechnology Park, Pune, 411057, India*

*^5^OmniActive Health Technologies, Wagle Estate, Thane, 400604, India*

*^6^OmniActive Health Technologies Inc, Morristown, NJ 07960, USA*

Running title: BCX pre-treatment protects the retina from LED injury

*Correspondence to: Kazim Sahin, DVM, Ph.D., F.A.C.N. Member of The Turkish Academy of Sciences Professor of Nutrition Veterinary Faculty Firat University 23119 Elazig, Turkey Phone:[+90-532-7473506](tel:+90%20532%20747%2035%2006) Phone:[+904242370000](tel:+90%20424%20237%2000%2000)/3938

Fax:[+904242388173](tel:+90%20424%20238%2081%2073), Email: [nsahinkm@yahoo.com](mailto:nsahinkm@yahoo.com), [ksahin@firat.edu.tr](mailto:ksahin@firat.edu.tr)

(A)


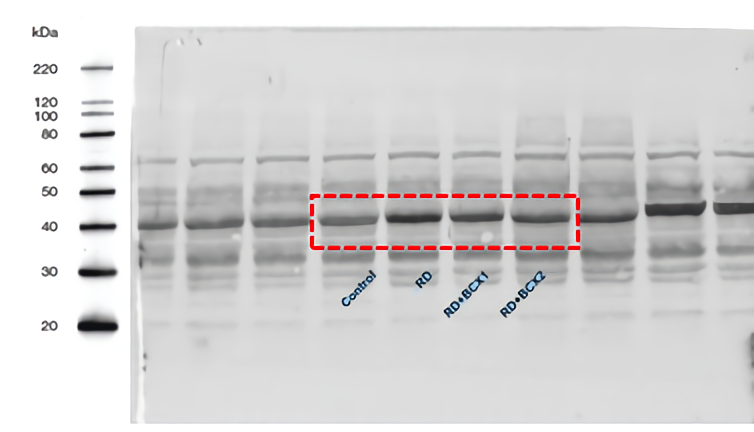


(B)


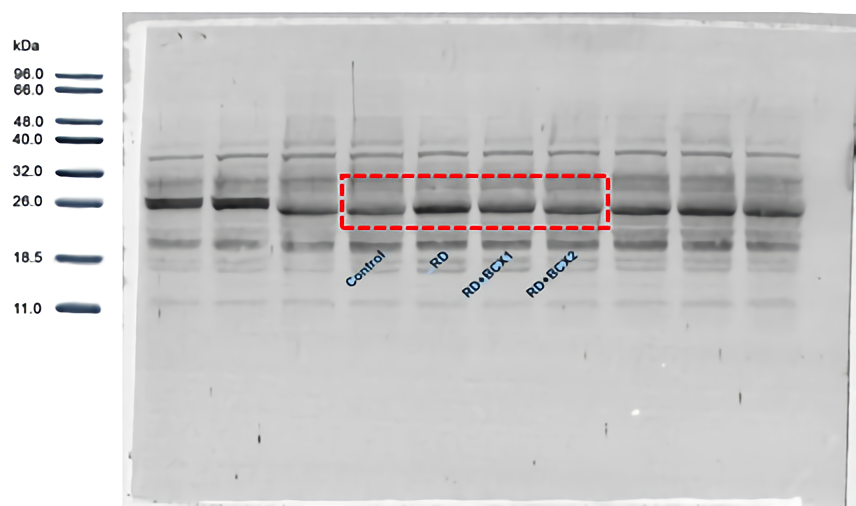


(C)

**
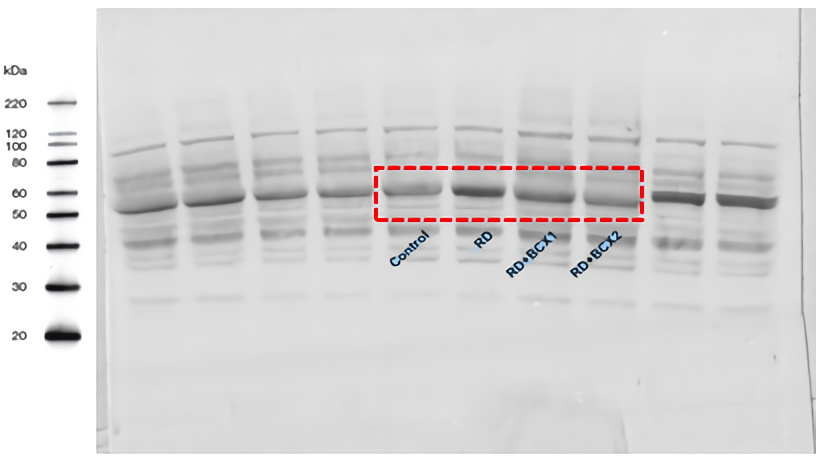
**

(D)

**
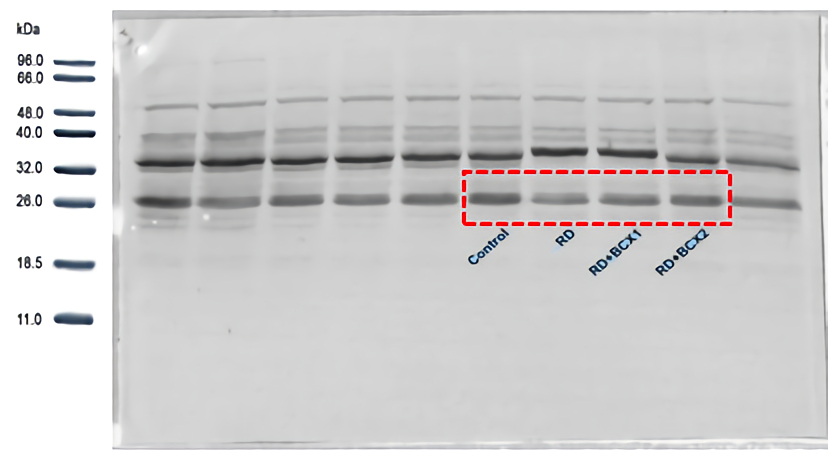
**

(E)


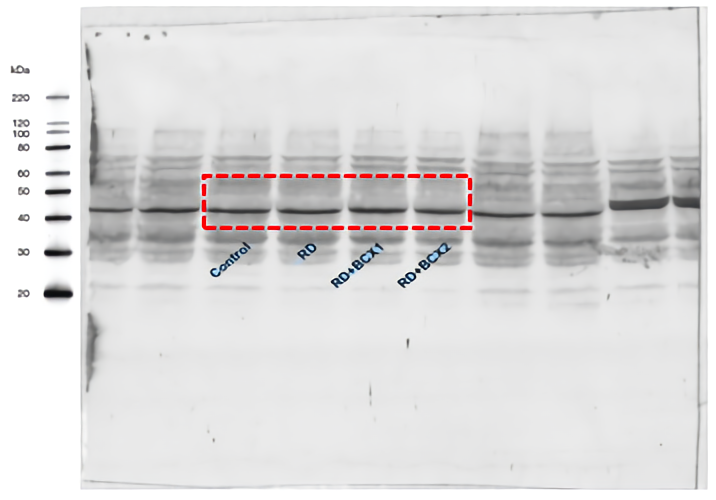


**Fig. 3. S1.** Full immunoblots related to Fig. 3 (IL-1β (A), IL-6 (B), NF-κβ (C), VEGF (D); β-Actin (E)). Each immunoblot is a representative of three independent experiments. Results shown in Fig.3 are delineated by red dotted rectangles. MW (in kDa) are indicated.

(A)


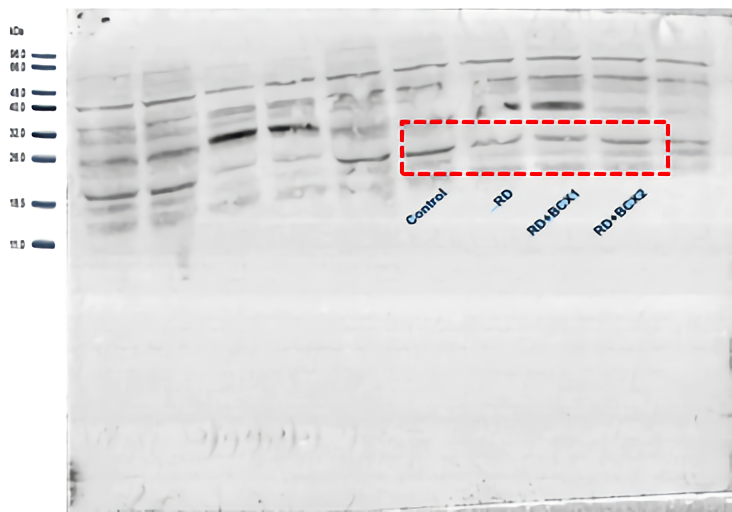


(B)
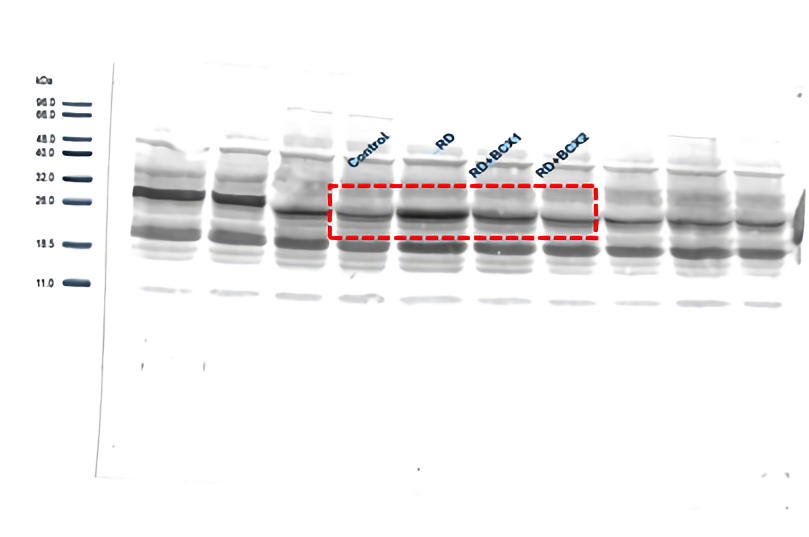


(C)


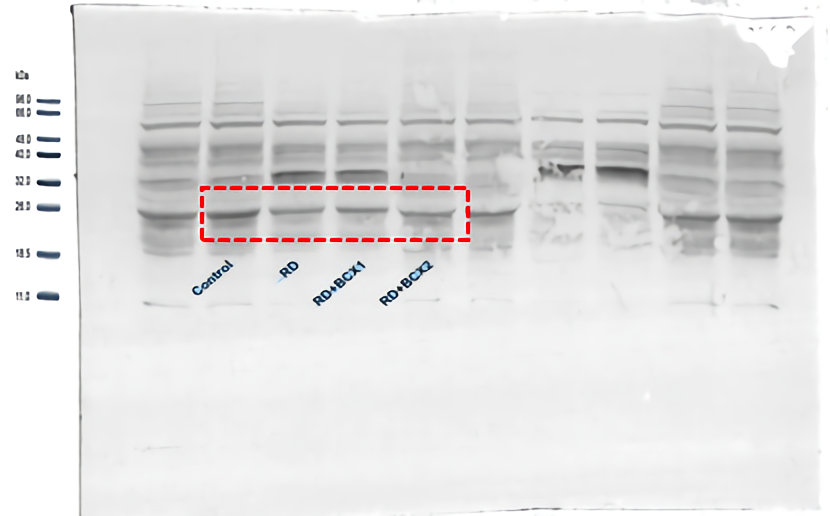


(D)


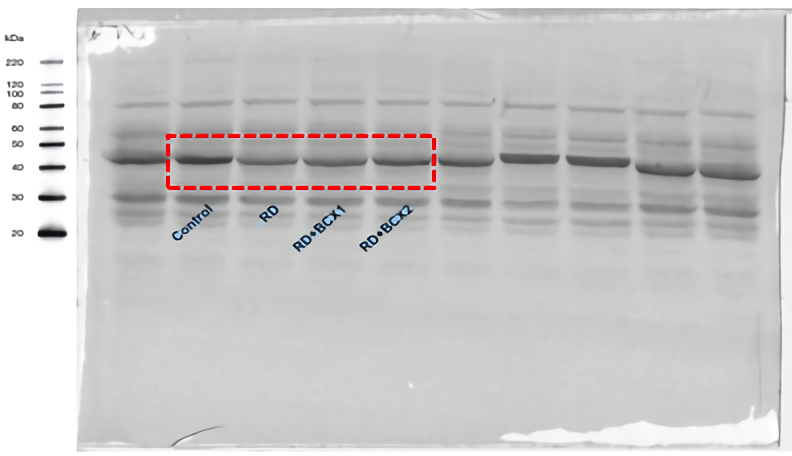


(E)


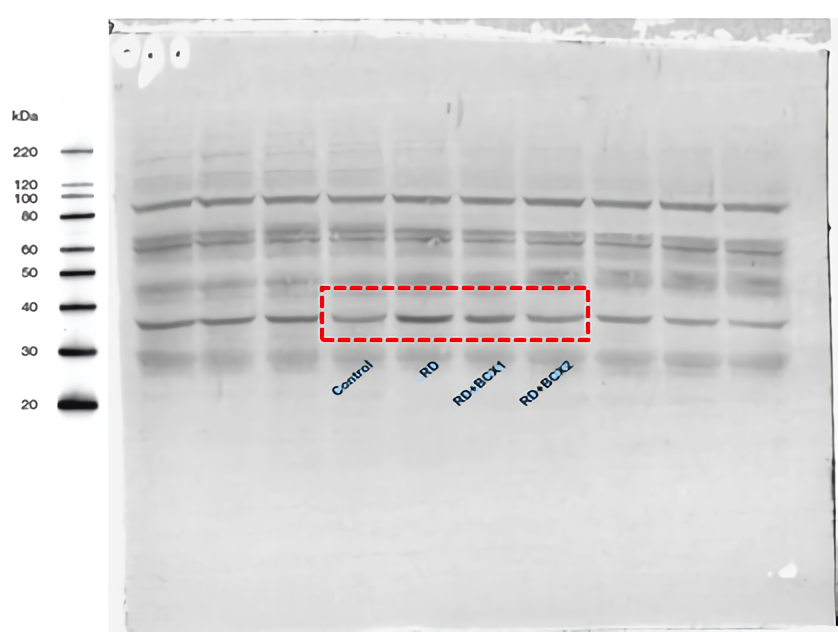


(F)
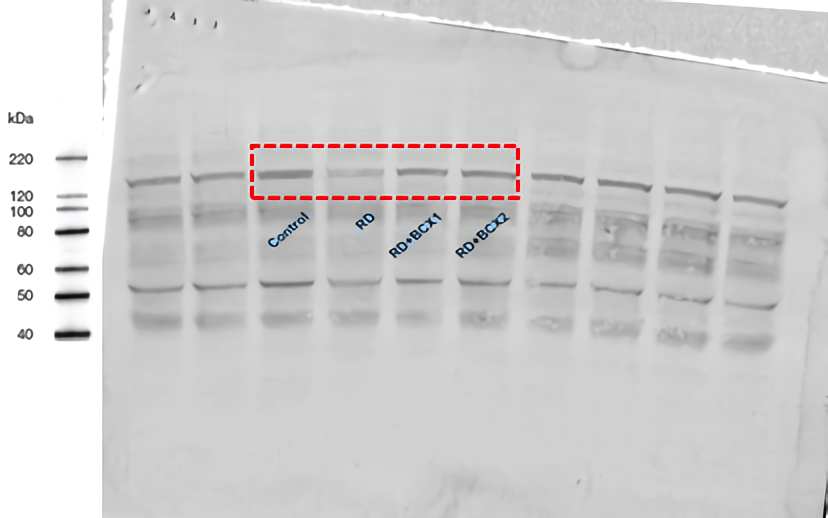


(G)


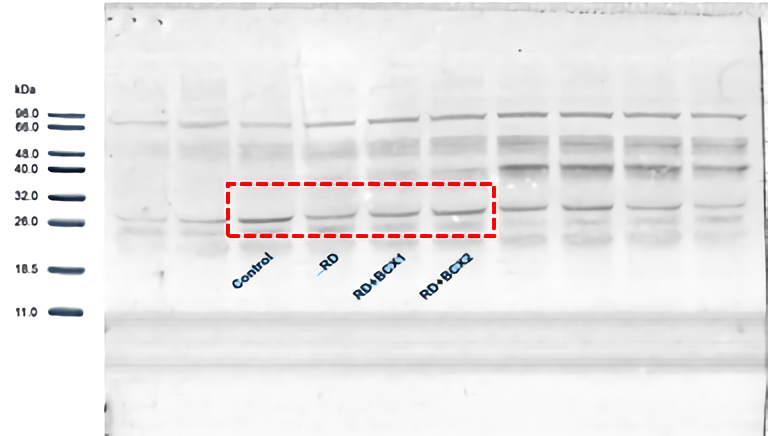


(H)


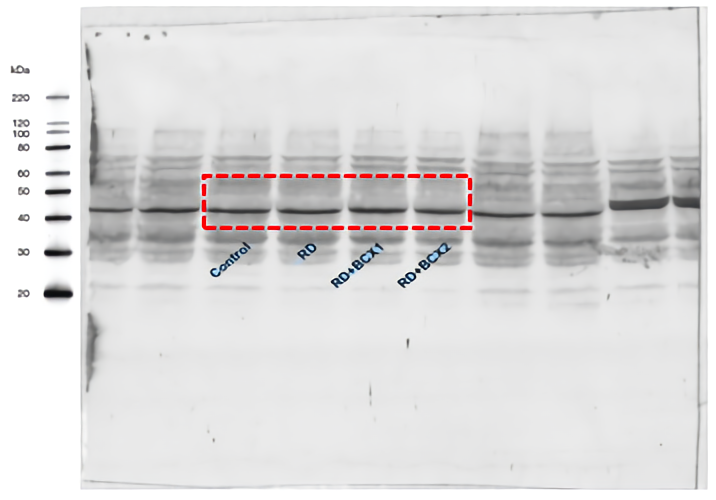


**Fig. 4. S1.** Full immunoblots related to Fig. 4 (Bax (A), Bcl-2 (B), Caspase-3 (C), Gap43 (D), GFAP (E), NCAM (F) HO-1 (G) and β-Actin (H)). Each immunoblot is a representative of three independent experiments. Results shown in Fig.3 are delineated by rectangles. MW (in kDa) are indicated.

(A)


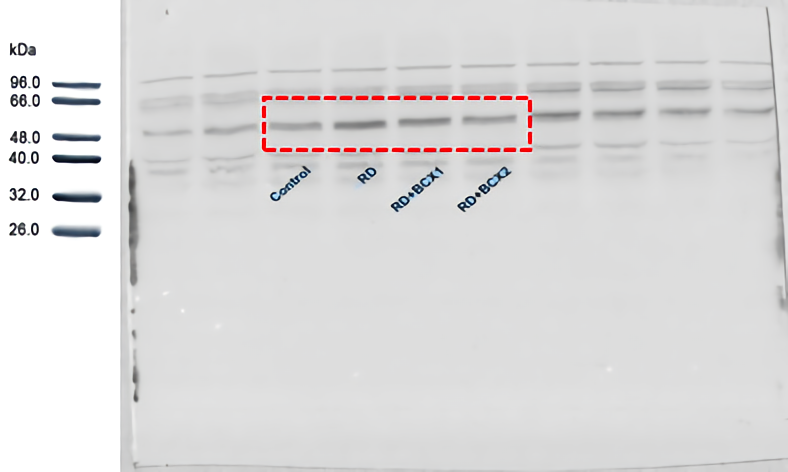


(B)


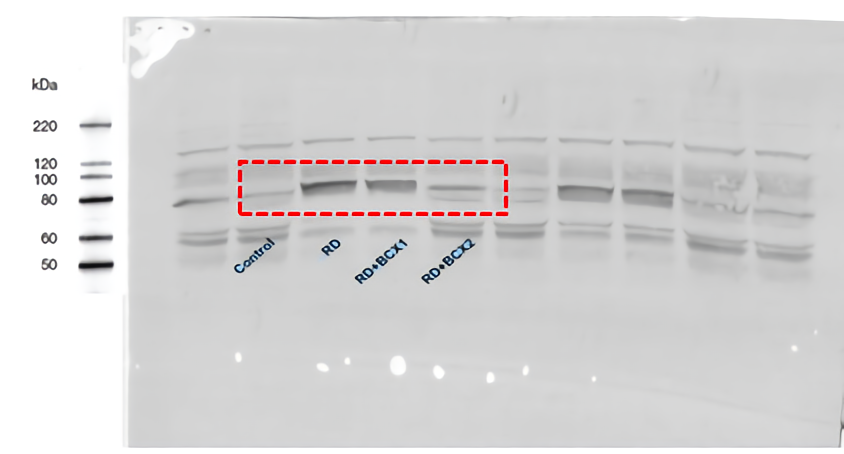


(C)


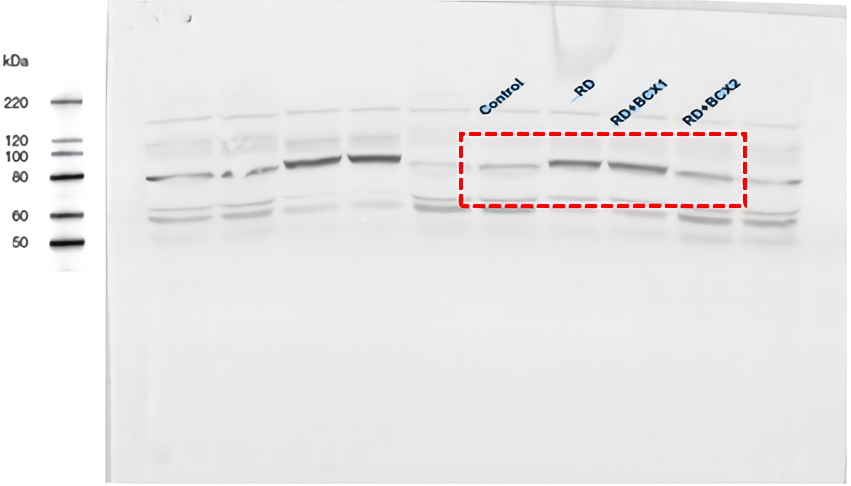


(D)


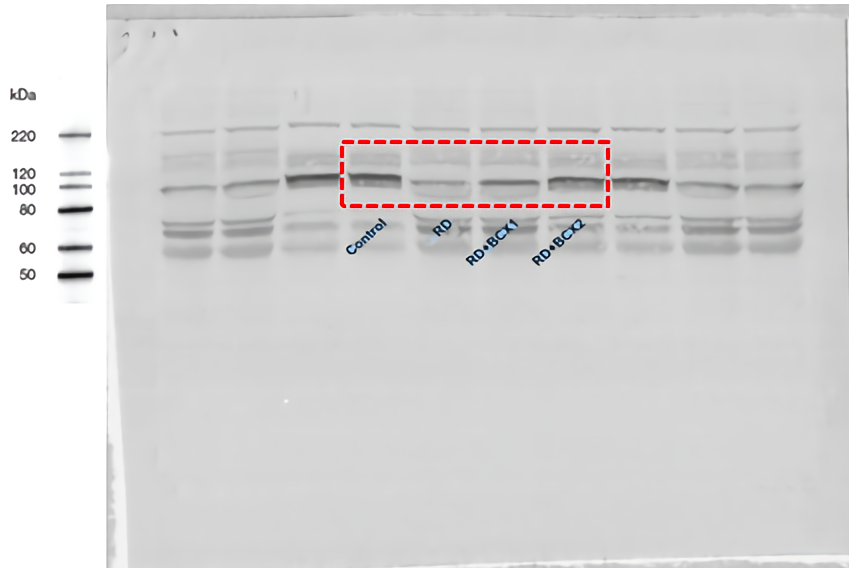


(E)
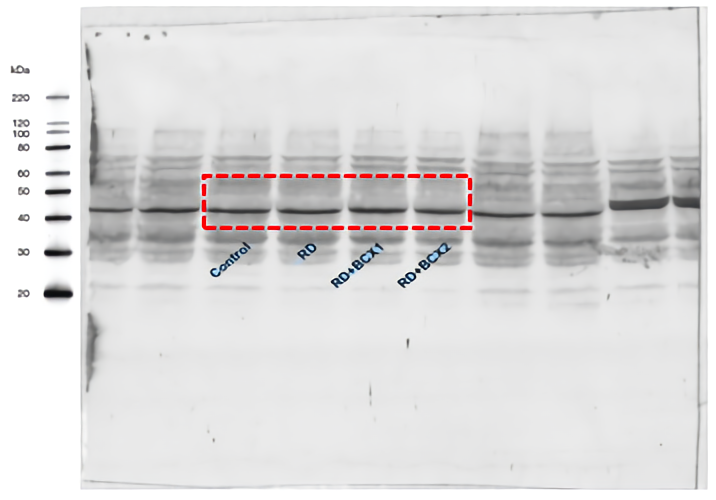


**Fig. 5. S1.** Full immunoblots related to Fig. 5 (ATF4 (A), ATF6 (B), Grp78 (C) and Grp94 (D) and β-Actin (E)). Each immunoblot is a representative of three independent experiments. Results shown in Fig.3 are delineated by rectangles. MW (in kDa) are indicated.
